# Time to Shift Focus from Oxidative Stress to Redox Regulation in COPD

**DOI:** 10.3390/antiox11020237

**Published:** 2022-01-26

**Authors:** Niki L. Reynaert

**Affiliations:** Department of Respiratory Medicine, NUTRIM School of Nutrition and Translational Research in Metabolism, Maastricht University Medical Center, 6229 ER Maastricht, The Netherlands; n.reynaert@maastrichtuniversity.nl; Tel.: +31-43-388-2270

Chronic obstructive pulmonary disease (COPD) is a non-communicable chronic disease that is top-ranking with respect to mortality and morbidity rates, posing an enormous burden on patients, caregivers and societies at large [1,2]. Although smoking is established as the main risk factor, the molecular and cellular mechanisms underlying the progressive deterioration of lung function remain to be unraveled; uncovering these mechanisms would at least enable halted disease progression using pharmacological interventions.

Oxidative stress has long been considered a key disease-driving mechanism in COPD. Research into the oxidant–antioxidant imbalance induced by smoking and its role in COPD has flourished since the late 1980s. As such, oxidative stress has been identified in lung tissue samples, as well as in samples of systemic bodily compartments in the form of end products of the oxidation of lipids, proteins and DNA (e.g., malondialdehyde, 4-hydroxynonenal, thiobarbituric acid-reactive substances, protein carbonyls and 8-hydroxy-2-deoxyguanosine) in multiple studies. Because they are relatively easy to measure, these oxidation products have been examined as non-invasive disease biomarkers [3,4].

In parallel, a myriad of studies have demonstrated suppressed antioxidant defense systems, which include both non-enzymatic and enzymatic antioxidants. A key role has been ascribed to defects in various components of the pathway that lead to the activation of the transcription factor that is key to the gene expression of antioxidant enzymes, namely nuclear factor erythroid 2-related factor 2 (Nrf2). Moreover, genetic susceptibility is conferred by single-nucleotide polymorphisms and other genetic variants in several antioxidant and detoxification genes themselves [3,4,5]. Given the combination of this failing antioxidant defense system and the enhanced burden put on it by smoke exposure itself and the reactive oxygen and reactive nitrogen species (ROS/RNS) derived from the chronically activated immune system, oxidative stress could be considered inevitable.

Although smoking cessation is still one of the most effective ways to delay disease progression at this point, smoke-related, exogenous ROS/RNS are likely not the paramount contributor to the progression of COPD, as smoking cessation does not halt lung function deterioration. Importantly, furthermore, a significant proportion of COPD patients does not have a smoking history [6]. Endogenous ROS/RNS derived from inflammation are likely more crucial given that current treatments also do not significantly affect inflammation or oxidative stress. In addition, disease exacerbations, which are key determinants of disease progression, further boost inflammation and oxidative stress [7].

In support of the theory that oxidative stress is a key driver of COPD, many of the studies on oxidative stress in COPD found a significant inverse correlation with lung function or disease severity. Furthermore, animal studies using the genetic manipulation of antioxidant systems confirm the contribution of the oxidant-to-antioxidant imbalance to the initiation and progression of COPD [8,9]. One caveat in these studies is that most animal models of COPD only mimic the emphysema phenotype of the disease. Direct and indirect damage to lung tissue, cells and its macromolecular components is considered the mechanism by which oxidative stress contributes to the disease. In addition to the defects in the antioxidant and detoxification systems, it should be noted that the cellular mechanisms present to restore oxidative damage to macromolecules also do not adequately function in COPD (DNA repair, mechanisms involved in controlling proteostasis, …). As such, oxidants have been shown to fuel inflammation, the protease-to-antiprotease imbalance, mucus metaplasia and mucin overexpression, and steroid resistance, and can be linked to the production of auto-antibodies, as reviewed in this Special Issue [3,4,5,10].

Following this growing evidence on the role of oxidative stress in COPD and initial success in animal models, clinical studies aimed at limiting oxidative stress levels by various generic pharmacological means were initiated; yet, to date, these have proven to be minimally effective [8,9]. Like for many new experimental drugs tested in COPD, the only effect that could be demonstrated was a decrease in the exacerbation frequency. As this is a key determinant of lung function deterioration, it should be regarded as a positive outcome [7], yet the working mechanism might not even be related to the antioxidant properties of the tested compounds [4,5].

The failure of antioxidant strategies in COPD has curbed the enthusiasm of many researchers and led the number of studies reporting on oxidative stress in COPD to reach a plateau for 8 years, averaging 147 publications per year (retrieved from Pubmed based on the search terms “oxidative” AND “stress” AND “COPD”, on 16 December 2021). This Special Issue on Redox Regulation in COPD hopes to reinvigorate research interest into the role of oxidative stress in COPD. All four excellent reviews offer perspectives on why antioxidant strategies have largely failed so far [3,4,5,10]. These include more recently obtained significant insight into the physiological functions of ROS and associated with this, the dose-dependent behavior elicited by ROS described as “hormesis”. These important aspects should be taken into account when attempting to combat oxidative stress, and the aim of interventions should thus be to restore redox homeostasis in order to preserve the physiological functions of ROS. Hence, the title of this Special Issue includes the term “redox regulation” instead of “oxidative stress”.

Importantly, in this context, the reviews highlight additional endogenous sources of ROS. In our own review, we focus on the beneficial effects of ROS signaling derived from various NADPH oxidases and their impairment in aging and COPD [4]. Inadvertently, these beneficial effects would have been impacted by non-discriminating antioxidant supplements. Endogenous sources, including mitochondria and NADPH oxidases, offer unique opportunities to finally target more specific sources of ROS, which would limit or boost a broad range of downstream effects of ROS and will therefore likely be more effective and selective strategies [4,10]. Moreover, additional antioxidant systems were discovered after the initial wave of interest into oxidative stress in COPD. In particular, the review by Kiyokawa et al. details our understanding of thioredoxin and discusses ways in which this redox system can be targeted and enhanced [5]. In addition, the review by Mizumura et al. emphasizes the potential contribution of more recently described modes of cell death to COPD, namely ferroptosis and necroptosis, which also offer more specific pharmacological targets connected to redox imbalances [10].

Redox regulation in COPD remains under-investigated compared to oxidative stress, only yielding an average of nine publications per year since 2013 (retrieved from Pubmed based on the search terms “redox” AND “regulation” AND “COPD”, on 16 December 2021), despite the promising studies and findings reviewed in this Special Issue. Through these reviews, we hope researchers in the redox field can be convinced to apply their knowledge and expertise to this top-ranked disease. Likewise, researchers already in the respiratory field should take note of these promising new research avenues so that much-needed steps forward can be taken in the treatment of COPD.

In doing so, there are some important considerations to take into account. First, in contrast to oxidative stress, it is much more challenging to measure reversible oxidations that are involved in redox regulation. Thorough biochemical expertise is required for the careful preparation, appropriate storage and analyses of biological samples. This is essential in order to preserve these oxidations, to prevent artefacts and confounding factors and to report reliable outcomes of studies. Secondly, interventions should focus on early disease because redox strategies in isolation will likely not restore or regenerate lung tissue that was lost or remodeled. Alternatively, redox-restoring strategies could be combined with regenerative approaches in more severe disease. Importantly, unwarranted side effects of such treatments can be expected, most notably tumorigenesis, in a population already at an increased risk for the development of lung cancer [11]. Careful monitoring and/or patient stratification based on defects in particular redox pathways should therefore be taken into account in clinical trials. On a positive note, redox imbalances and redox dysregulation contribute not only to local pulmonary disease in COPD, but also to co-morbidities. As such, studies into novel therapeutic strategies and biomarkers should take additional organs and co-existing diseases into consideration. Lastly, the free radical theory of aging is also slowly being replaced by the more intricate view on the role of redox regulation in aging [4,5]. Compounds that arise from the global quest to extend the healthy life-span can prove to be beneficial in the treatment of COPD as well, as it is now viewed as a syndrome of accelerated aging [12].
